# Strontium and Zinc Substitution in β-Tricalcium Phosphate: An X-ray Diffraction, Solid State NMR and ATR-FTIR Study

**DOI:** 10.3390/jfb10020020

**Published:** 2019-05-05

**Authors:** Elisa Boanini, Massimo Gazzano, Carlo Nervi, Michele R. Chierotti, Katia Rubini, Roberto Gobetto, Adriana Bigi

**Affiliations:** 1 Department of Chemistry “Giacomo Ciamician”, Alma Mater Studiorum-University of Bologna, 40126 Bologna, Italy; elisa.boanini@unibo.it (E.B.); katia.rubini@unibo.it (K.R.); adriana.bigi@unibo.it (A.B.); 2 ISOF-CNR, via Gobetti 101, 40129 Bologna, Italy; 3 Department of Chemistry, University of Torino, via P. Giuria 7, 10125 Torino, Italy; carlo.nervi@unito.it (C.N.); michele.chierotti@unito.it (M.R.C.); roberto.gobetto@unito.it (R.G.)

**Keywords:** β-tricalcium phosphate, zinc, strontium, ionic substitutions, Rietveld refinement, solid-state NMR, ATR-FTIR

## Abstract

β-tricalcium phosphate (β-TCP) is one of the most common bioceramics, widely applied in bone cements and implants. Herein we synthesized β-TCP by solid state reaction in the presence of increasing amounts of two biologically active ions, namely strontium and zinc, in order to clarify the structural modifications induced by ionic substitution. The results of X-ray diffraction analysis indicate that zinc can substitute for calcium into a β-TCP structure up to about 10 at% inducing a reduction of the cell parameters, whereas the substitution occurs up to about 80 at% in the case of strontium, which provokes a linear increase of the lattice constants, and a slight modification into a more symmetric structure. Rietveld refinements and solid-state ^31^P NMR spectra demonstrate that the octahedral Ca(5) is the site of β-TCP preferred by the small zinc ion. ATR-FTIR results indicate that zinc substitution provokes a disorder of β-TCP structure. At variance with the behavior of zinc, strontium completely avoids Ca(5) site even at high concentration, whereas it exhibits a clear preference for Ca(4) site. The infrared absorption bands of β-TCP show a general shift towards lower wavenumbers on increasing strontium content. Particularly significant is the shift of the infrared symmetric stretching band at 943 cm^−1^ due to P(1), that is the phosphate more involved in Ca(4) coordination, which further supports the occupancy preference of strontium.

## 1. Introduction

β-tricalcium phosphate, β-Ca_3_(PO_4_)_2_ (β-TCP) is the stable polymorph of tricalcium phosphate at temperatures lower than 1125 °C. At higher temperatures, it undergoes a thermal transition into the α form [1]. β-TCP is synthesized by solid state reaction at high temperature, alternatively it can be prepared by thermal transition of other calcium phosphates. Although this phase is not present in physiologically calcified biological tissues, it has been found in pathological calcifications and it can be obtained as a product of thermal conversion of the poorly crystalline hydroxyapatite (HA), which constitutes the inorganic phase of bone [2]. Actually, β-TCP both alone and in combination with synthetic HA, is widely applied as a biomaterial for the treatment of defects of the skeletal system [3]. 

The properties, as well as the relative stability, of calcium phosphates can be modified by functionalization with foreign ions [4]. The number of studies on ionic substitution into HA structure is particularly relevant, due to its high flexibility, which supports a great variety of cationic, as well as anionic, substitutions [4,5,6,7,8]. 

**Figure 1:**
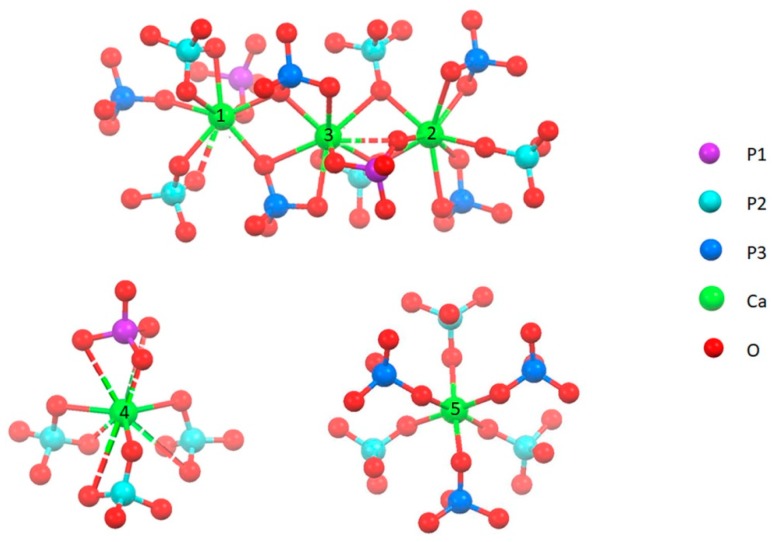
Coordination geometry of the five different cation sites of β-TCP [15]. Bonds at 3.0 ± 0.1 Å length are dotted.

The rhombohedral structure of β-TCP, space group R3c, is without any doubt less flexible than HA lattice; however, it can host several ionic replacements, especially the replacement of calcium ions with bivalent cations [9,10,11,12,13,14]. Calcium ions in β-TCP occupy five different cation sites: Ca(1), Ca(2) and Ca(3) are on general positions with eight to nine coordinated oxygens, whereas both Ca(4) and Ca(5) are on special positions, with an effective multiplicity of 1/3 of the other cation sites. Ca(5) exhibits an approximately octahedral coordination, Ca(4) site is only half occupied and exhibits a quite distorted nine coordination (Figure 1) [15]. Phosphorus exhibits three crystallographically different positions, with the multiplicity of P(1) equal to 1/3 of those of P(2) and P(3), since it is on a special position.

In this paper we investigated the structural modifications induced on β-TCP structure by functionalization with two bivalent cations, namely strontium and zinc. Both these ions are of great biological interest: strontium has been reported to display a beneficial action in the treatment of bone diseases characterized by abnormally high mass resorption, such as osteoporosis [16,17]; zinc has been shown to inhibit osteoclast proliferation [18] and it has been reported to display antibacterial properties [19]. Moreover, their beneficial effect on bone cells has also been observed when coupled to β-TCP [20,21,22,23,24]. In particular, strontium-substituted β-TCP (SrTCP) added with mesenchymal stem cells has been shown to improve bone formation and fusion across the transverse processes, both in osteoporotic and non-osteoporotic animal models [25]. The possible substitution of these two cations into β-TCP structure has been previously investigated. Heat treatment of poorly crystalline HA synthesized in the presence of zinc produced Zn substituted β-TCP (ZnTCP) up to a Zn content of about 9 at% [26]. The results of Rietveld refinement indicated a linear decrease of the lattice constant and a preference of Zinc for the Ca(5) site. Evidences of preference for the octahedral Ca(5) site were obtained also by combination of near-edge X-ray absorption fine structure (NEXAFS) measurements and *ab-initio* calculations with X-ray refinement of ZnTCP synthesized by solid state reaction up to a zinc content of about 3.3 at% [27]. These data are in agreement with the results of a study of first-principles calculations which indicated that Ca(4) and Ca(5) are the favored substitution sites of divalent cations: the lower coordination number and bond lengths of Ca(5), are preferred by smaller sized cations such as Zn and Mg, whereas larger-sized ones, such as Sr and Ba, favor the Ca(4) site [28]. On the other hand, on the basis of the refinement results of zinc doped biphasic calcium phosphate (BCP) Gomes et al. (2011) [29] concluded that Zn is incorporated into β-TCP structure by substituting calcium ions both in Ca(5) and in Ca(4) sites. Strontium substitution to calcium into β-TCP has been obtained by thermal treatment of calcium deficient apatite up to 20 at% and by solid state reaction up to 80 at% [9,30]. However, detailed structural analysis on strontium substituted β-TCP (SrTCP) was performed only up to a Sr substitution of about 33 at% and indicated a preferential occupation of the Ca(4) site [31,32].

Herein, we investigated the substitution of zinc and strontium to calcium into β-TCP structure by comparing solid-state NMR, ATR-FTIR and Rietveld refinement data of samples at different degree of cationic substitution.

## 2. Materials and Methods

### 2.1. Synthesis

β-TCP was obtained by solid-state reaction of CaCO_3_ and CaHPO_4_·2H_2_O (DCPD) in the molar ratio of 1:2 at 1000 °C for 12 h. The solid product was ground into a mortar before being submitted to further heat treatment.

In particular, two different materials were prepared following the procedure reported above: one was prepared using commercial DCPD (Sigma-Aldrich, Milano, Italy) and labeled as β-TCP-C; the other one (labeled just as β-TCP) was synthesized using freshly-prepared DCPD. The synthesis of DCPD was carried out using 600 mL of a phosphate solution containing 0.08 mol of Na_2_HPO_4_·12H_2_O (Carlo Erba, Milano, Italy) and 0.08 mol of NaH_2_PO_4_·H_2_O (Carlo Erba), pH 4 adjusted with glacial CH_3_COOH. The solution was heated at 37 °C and 200 mL of solution containing 0.16 mol of Ca(CH_3_COO)_2_·H_2_O (Carlo Erba) was added drop-wise over a period of about 60 min, under continuous stirring. Afterwards the precipitate was stored in contact with the mother solution for 10 min, filtered, repeatedly washed with bidistilled water and dried at 37 °C.

For the preparation of Sr-substituted β-TCP, α-Sr_3_(PO_4_)_2_ was also prepared by solid-state reaction at 1200 °C of SrCO_3_ and (NH_4_)_2_HPO_4_ in the molar ratio of 3:2 for 12 h. Sr-substituted β-TCP samples with a Sr content of 10, 20, 40, 60 and 80 atom% (with respect to total cations Ca + Sr) were prepared by heat treatment of appropriate stoichiometric mixture of β-TCP and α-Sr_3_(PO_4_)_2_ at 1000 °C for 12 h. Samples were labeled 10SrTCP, 20SrTCP, 40SrTCP, 60SrTCP and 80SrTCP, respectively. For the preparation of Zn-substituted β-TCP, α-Zn_3_(PO_4_)_2_ was also prepared by solid-state reaction at 800 °C of (ZnCO_3_)_2_[Zn(OH)_2_]_3_ and (NH_4_)H_2_PO_4_ in the molar ratio of 3:10 for 12 h. Zn-substituted β-TCP samples with a Zn content of 5, 10 and 15 atom% (with respect to total cations Ca + Zn) were prepared by heat treatment of appropriate stoichiometric mixture of β-TCP and α-Zn_3_(PO_4_)_2_ at 1000 °C for 8 h. Samples were labeled 5ZnTCP, 10ZnTCP and 15ZnTCP, respectively.

### 2.2. Characterization

X-ray diffraction analysis was carried out by means of a PANalytical X’Pert PRO powder diffractometer equipped with a fast X’Celerator detector. Cu Kα radiation was used (40 mA, 40 kV). The 2θ range was from 10° to 60° with a step size of 0.05° and time/step of 150 s. Data used for structural investigation were collected in the 2θ range 10°–100° with a 0.026° step and counting time of 500 s/step.

MAUD program (Material Analysis Using Diffraction) [33] was used for structural refinements employing scattering factors for Ca^2+^, Sr^2+^, Zn^2+^ and O^2−^ ions, as well as for phosphorus atom. The background was fitted as a polynomial function. The process started with the refinement of scale factor, background coefficients and 2θ shift. In the further cycles cell axes, peak widths, their dependence on 2θ, asymmetry and Gaussianity fraction of the peaks were kept as free variables. Finally the structural parameters (occupancy factors and coordinates) were refined using rigid body constrain for phosphate tetrahedra. The reported lattice parameters resulted from refinements.

Solid-state NMR spectra were acquired with a Jeol ECZR 600 instrument, operating at 242.95 MHz for the ^31^P nucleus. Powder samples were packed into cylindrical zirconia rotors with a 3.2 mm o.d. and a 60 µL volume and spun at 20 kHz. A certain amount of sample was collected and used without further preparations from all samples to fill the rotor. ^31^P MAS spectra were acquired at room temperature for all samples. The spectra were collected using a with the direct excitation pulse sequence with a 90° ^31^P pulse of 1.3 µs, an optimized recycle delay of 1800 s and a number of scans between 4–8, depending on the sample. The ^31^P chemical shift scale was calibrated through a 85% H_3_PO_4_ solution as external standard.

For infrared absorption analysis in attenuated total reflection (ATR) mode, samples were analyzed using a Bruker ALPHA FT-IR spectrometer equipped with a diamond unit, to collect 64 scans in the range 400–4000 cm^−1^ at a resolution of 4 cm^−1^. Data analysis was operated with OPUS software.

Morphological investigation was performed using a Hitachi S-2400 scanning electron microscope operating at 15 kV. Sputter-coating with gold was performed before examination.

## 3. Results

**Figure 2:**
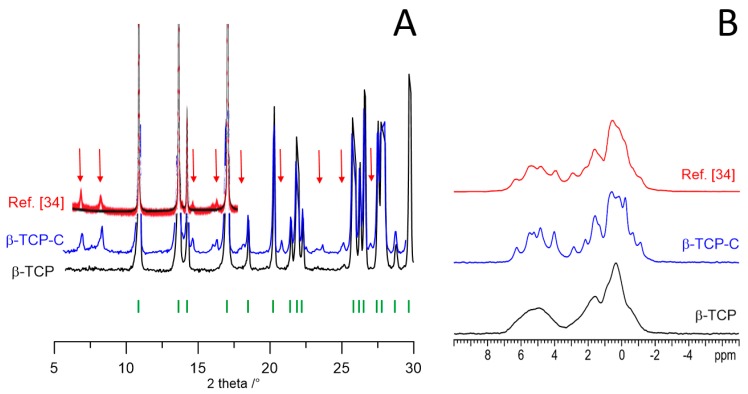
(**A**) XRD patterns and (**B**) ^31^P (242.95 MHz) MAS solid-state NMR spectra of β-TCP (black), β-TCP-C (blue) and β-TCP data from Mellier et al. [34] (red). The reflection markers for rhombohedral β-TCP are reported as vertical bars (green). Red arrows highlight peaks not belonging to rhombohedral β-TCP. Adapted with permission from reference [34]. Copyright (2011) American Chemical Society.

The solid-state synthesis of β-TCP yielded two different products depending on the type of CaHPO_4_·2H_2_O (DCPD) utilized as reagent. The X-ray diffraction pattern of the material obtained using freshly synthesized DCPD (β-TCP) is compared with that of the product of the synthesis performed using commercial DCPD (β-TCP-C) in Figure 2. Although most of the peaks are the same in the two patterns, β-TCP-C displays several peaks of low intensity which are not characteristic of pure β-TCP, as proved by comparison with calculated pattern (Figure S1). On the other hand, the ^31^P MAS NMR spectrum of β-TCP-C exhibits a much higher number of narrow and slightly overlapping resonances than that of β-TCP (Figure 2).

Similar peculiar XRD pattern and NMR spectrum (also shown in Figure 2 for comparison) were previously recorded by Mellier et al. (2011) [34], who ascribed the NMR resonances to at least 16 inequivalent P sites and suggested a lower symmetry superstructure on the basis of the presence of two weak low angle reflections not allowed by R3c symmetry. The large number and narrow line width of the resonances agree with a high ordering of the vacancies within the β-TCP structure rather than a random distribution, since the latter would result in significantly broad ^31^P signals. Mellier et al. (2011) [34] also performed a preliminary assignment of the ^31^P isotropic chemical shifts by DFT calculations using the GIPAW method [35,36].

However, the XRD pattern recorded from β-TCP-C, as well as that reported by Mellier et al. [34] although not highlighted by the Authors, shows the presence of a number of further weak reflections in addition to the two weak low angle reflections, which leads to suggest that these materials contain further, not identified, crystalline phases. Thus, XRD and NMR data of β-TCP-C seem to lead to different conclusions. In order to bypass this apparent contradiction and highlight just the influence of ionic substitution on β-TCP structure, we carried out all the syntheses of the different samples using DCPD synthesized in our lab which yields β-TCP characterized by a XRD pattern where all the peaks correspond to those of the calculated one on the basis of rhomboedral structure.

**Figure 3:**
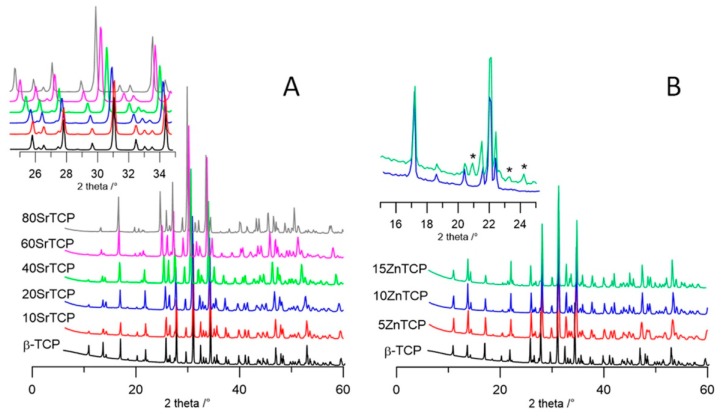
XRD patterns of strontium substituted (**A**) and zinc substituted; (**B**) samples compared with unsubstituted β-TCP. The peaks not corresponding to those characteristic of β-TCP are indicated with *.

The powder X-ray pattern of products synthesized in the presence of different amounts of Sr^2+^ and Zn^2+^ are reported in Figure 3A,B, respectively. In agreement with previous data [9], the patterns of Sr-TCP indicate the presence of a unique crystalline phase up to a Sr^2+^ content of 80 at%.

Moreover, the evident shift of the reflections towards lower angles on increasing the Sr concentration (Figure 3A inset) are in agreement with the substitution of the bigger cation (radius 0.118 nm) to the smaller Ca (0.100 nm). At variance, the amount of Zn that can be hosted by the structure of β-TCP is noticeably lower: inset in Figure 3B shows that only the patterns of the samples synthesized in the presence of 5 and 10 Zn at% are in agreement with the presence of a unique crystalline phase, whereas at a higher Zn concentration further peaks indicate the presence of a secondary phase.

**Figure 4:**
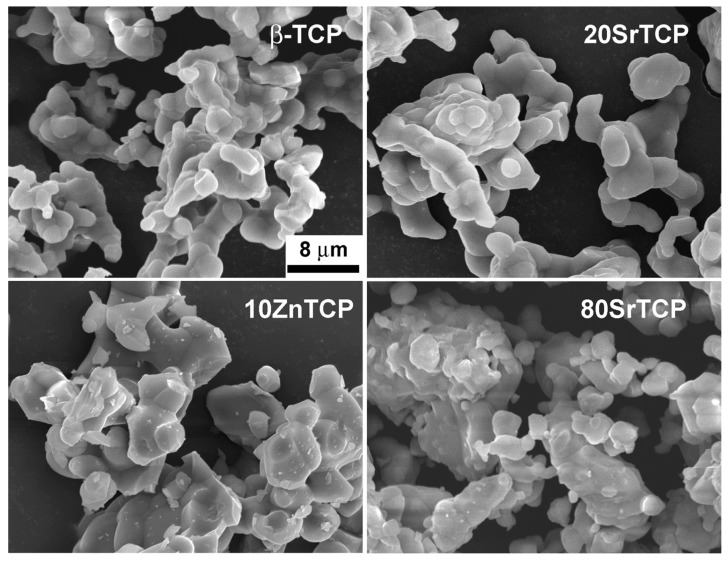
SEM images of β-TCP, 20SrTCP, 80SrTCP and 10ZnTCP show the characteristic morphology of a solid-state reaction product.

Scanning electron microscopy image of pure β-TCP shows the characteristic micrometric particles morphology with rounded edges, which does not seem to be significantly affected by the presence of a low amount of Sr (Figure 4). The products synthesized at increasing Sr content, as well as in the presence of Zn, appear to be constituted of more dense blocks, which however retain the characteristic morphology of a solid-state reaction product.

### 3.1. Structural Analysis

The structural refinements were performed by the Rietveld method [37] starting from the atomic position set of β-TCP in space group R3c (n. 161) [15]. Since the asymmetric unit contains five calcium, three phosphorus and ten oxygen atoms, in order to limit the number of free variables we used a rigid body model with constraints to maintain the same geometry of phosphate tetrahedra as in pure β-TCP. Phosphorus atom positions were kept free to move in accordance with the symmetry rules of the crystal system.

**Figure 5:**
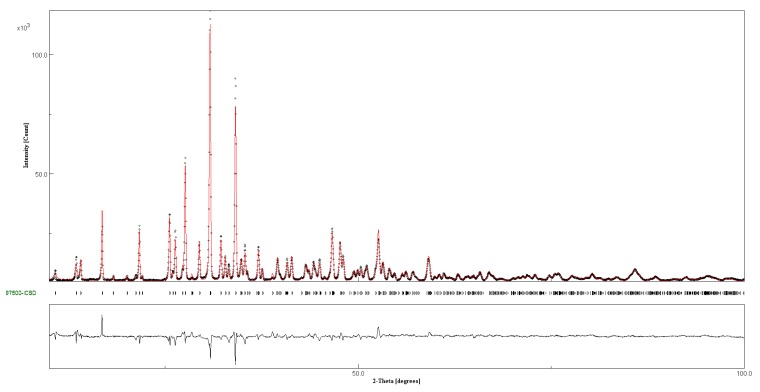
Comparison of the observed (dots) and calculated (red) patterns of 20SrTCP. At the bottom reflection markers and curve difference.

Thermal parameters were fixed at the values of β-TCP [15] because of their high correlation with occupancy factors (OF), but an overall thermal parameter factor was allowed to vary. The sum of the OF of calcium and of the substituent ion in each metal (M) site was imposed to unity (with the exception of site M(4) which was 0.43 in accordance with β-TCP structure [15]). No constraint was imposed on the overall metal atoms content and no attempt was made to differentiate the calcium positions from the substituent metal ones. A graphical plot of the refinement of sample 20SrTCP is reported in Figure 5 as an example, whereas the most relevant structural parameters are reported in Table 1 and Table 2. Full set of calculated vs. measured plots are reported in Supplementary Material (Figure S2), whereas the structural data of SrTCP and ZnTCP samples are deposited in ICSD files (1905508 10SrTCP, 1905509 10ZnTCP, 1905510 60SrTCP, 1905511 40SrTCP, 1905512 80SrTCP, 1905513 20SrTCP, 1905514 5ZnTCP).

**Figure 6:**
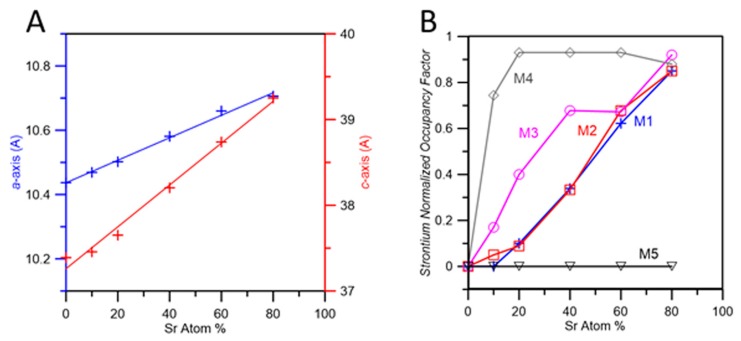
(**A**) Plot of *a*- and *c*-axis as a function of strontium content; (**B**) Values of normalized occupancy factors of each of the five metal position as a function of Sr atom content. The values for sample 80SrTCP values are obtained from the refinement performed using the β’-TCP structure as the starting model.

The variation of the unit cell parameters as a function of Sr content is reported in Figure 6A. The trend is strictly linear and fits well with previously reported data [9,38]. The overall strontium contents resulting from the refinements with no imposed constraints are very close to the analytical ones. However, agreement indexes (Rwp, Table 2) show a worsening as strontium content increases, and the Rwp value obtained for 80SrTCP is particularly high. In order to improve the fit, we refined 80SrTCP using a somewhat different starting model, namely the structure of β’-TCP. This structure (*R-*3*m*) was reported to occur as a thermal phase transition of β-TCP promoted by strontium [32]. It is topologically similar to β-TCP, the main difference being the presence of a symmetry center that implies a unit cell with half *c*-axis and volume. In β’-TCP, M(1) and M(2) sites, as well as P(2) and P(3) tetrahedra, are equivalent to each other (M(1) and P(2) respectively); M(3) is slightly displaced from the symmetry element and split into M(31) and M(32), each of which with occupancy of ¼, and M(4) has occupancy of ¼ [32]. Refinement of 80SrTCP using as a model the structure of β’-TCP indeed gives a better fitting, as shown by the data reported in Table 2 and in Figure S3. In contrast, samples at lower strontium content cannot be described using β’-TCP structure since they present some XRD peaks which are characteristic of β-TCP and cannot be indexed using the β’-TCP unit cell setting, as shown in Figure S4.

**Figure 7:**
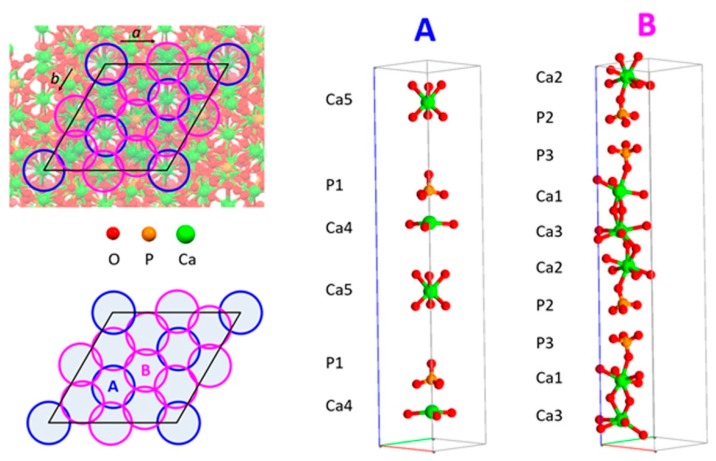
**Left**, views of the β-TCP unit cell down the *c*-axis. **Right**, a view of the atoms that characterize A- and B-type ‘columns’.

The normalized OF of SrTCP samples are reported in Figure 6B as a function of strontium content. The choice to plot normalized factors is obliged by the big difference in the multiplicity of M(1), M(2) and M(3) (multiplicity = 18) in comparison to that of M(4) and M(5) sites (multiplicity = 6) that would mask the relative cation distribution if plotted as absolute values. Results of powder fitting refinements indicate that at low degree of substitution strontium displays the highest OF in M(4) site, followed by M(3) site; as the overall substitution degree increases, the filling of sites M(1) and M(2) become important, too. It is worth mentioning that in the whole range of investigated composition (strontium up to 80 at%), site M(5) is not occupied by Sr atoms. The ‘filling’ of calcium sites by strontium starting from M(4) and M(3) is also due to a geometrical reason. β-TCP structure can be described as a regular assembly of two kinds of columns in which Ca atoms and phosphate tetrahedra are stacked. As shown in Figure 7, the unit cell view down *c*-axis allows to identify A-type columns filled with Ca(4), Ca(5) and P(1) whereas B-type columns contain Ca(1), Ca(2) Ca(3) and P(2) and P(3). A-type columns are surrounded only by B-type ones. At first strontium enters into Ca(4), causing a significant expansion of *c*-axis. Further strontium substitution for calcium is better accommodated in the other column to compensate the increment along the *c*-axis.

Moreover, the preference of strontium for sites M(4) and M(3) can be ascribed to the most favorable distances found in these sites, which can accommodate bigger ions than calcium. Mean Ca-O distances in β-TCP are: Ca(1)-O 2.505 Å, Ca(2)-O 2.487 Å, Ca(3)-O 2.601 Å, Ca(4)-O 2.936 Å, Ca(5)-O 2.263 Å. Ca-O environments exhibit the longest mean distance in site M(4).

Mean M(3)-O distance is second just to M(4)-O and any further increments in Sr content can be easily accommodated here, also due to its higher storage ability than M(4): the unit cell contains less than 3 M(4) type atoms vs. 18 atoms of the M(3) type. On the other hand, location of strontium in site M(5) is strongly unfavorable since the mean M(5)-O distance is the shortest. Furthermore, the range of M-O bond values in each site should also play a role: M(4) shows the shorter contact at 2.53 Å, M(5) at 2.21 Å and all the other sites at values greater than 2.30 Å [15]. These data agree with the preference of strontium for site M(4) reported previously on β-TCP samples containing small amount of strontium [31,39], and demonstrate that the preference is maintained also at much higher strontium content. In fact, the results obtained for the sample 80SrTCP indicate the same preference both when the refinement is performed using the structure of β-TCP [15] and that of β’-TCP [32].

The Zn atom, due to its smaller ionic radius (0.075 nm) than calcium, shows a different behavior. The results of the structural refinements (Figure S5, Table 3) show that zinc is distributed over all the sites in ZnTCP at a low substitution degree (5%), but M(5) becomes the preferred site when zinc content increases (10%). The octahedral coordination geometry and short distances of M(5) are particularly suitable for the little zinc ion. Probably due also to the limited substitution range, ZnTCP does not present any discontinuity in cell parameters, as previously reported for MgTCP [40], where magnesium was found to fill both M(4) and M(5) sites.

### 3.2. Solid-State ^31^P MAS NMR

The ^31^P isotropic chemical shift is very sensitive to local structural modifications, thus providing a reliable probe for this class of samples. For the system under investigation the Zn and Sr substitutions were followed by ^31^P MAS NMR.

**Figure 8:**
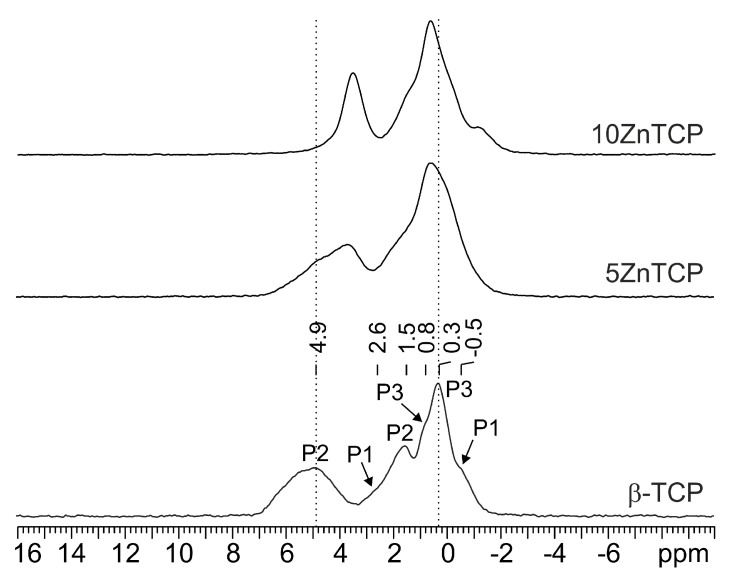
^31^P (242.95 MHz) MAS spectra of β-TCP (with relevant assignment), 5ZnTCP and 10ZnTCP, acquired at room temperature with a spinning speed of 20 kHz.

The ^31^P MAS NMR spectrum of pure β-TCP (Figure 8 bottom) is characterized by three main broad resonances at 4.9 (P2), 1.5 (P2) and 0.3 (P3) ppm and three shoulders at 2.6, 0.8 and −0.5 ppm assigned to P1, P3 and P1, respectively. The spectrum strongly resembles that reported by Obadia et al. (2006) [41] for the sample named TCPPrecip obtained by precipitation of a Ca(OH)_2_ solution by the addition of aqueous H_3_PO_4_, followed by calcination.

**Figure 9:**
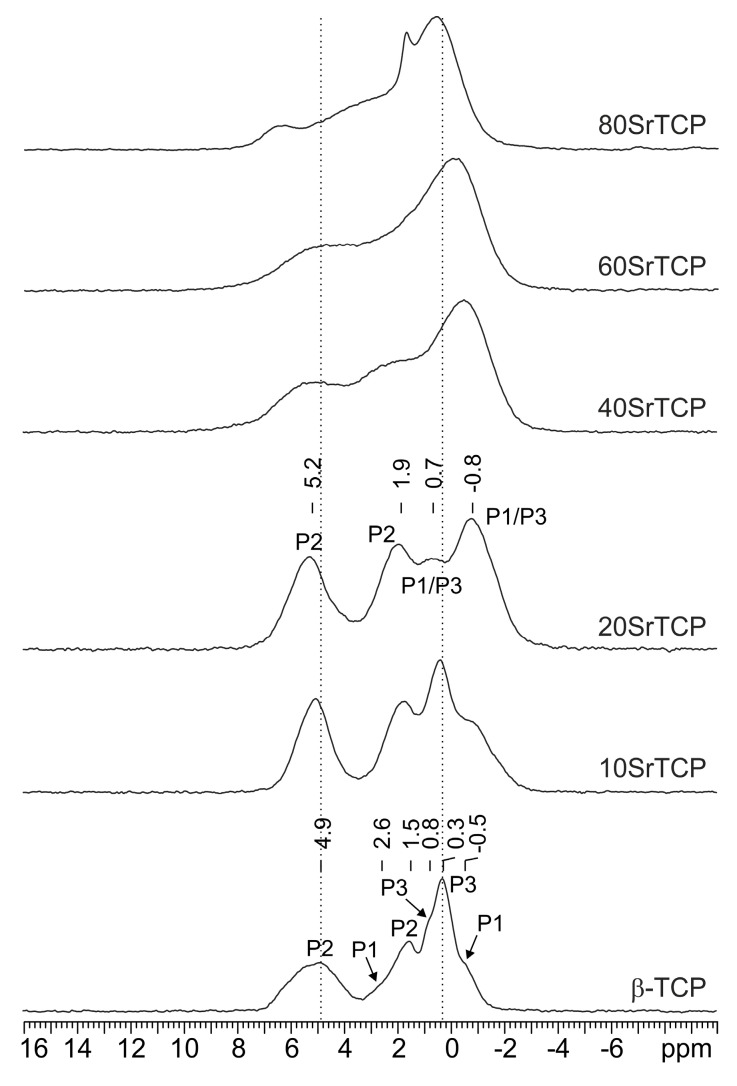
^31^P (242.95 MHz) MAS spectra of β-TCP (with relevant assignment), 10SrTCP, 20SrTCP (with relevant assignment), 40SrTCP, 60SrTCP and 80SrTCP acquired at room temperature with a spinning speed of 20 kHz.

Even for a very low substitution of Ca with Zn or Sr in the β-TCP structure, the ^31^P resonances change in terms of chemical shift and line width (Figure 8 and Figure 9). In particular, the broadening observed in the substituted samples suggests a random substitution of the foreign ions for calcium.

As discussed in Section 3.1, XRD data indicate that Zn substitution takes place preferentially on the M(5) site, which is surrounded by the P2 and P3 phosphorus sites (Figure 1). On the other hand, Sr exhibits a clear preference for an M(4) site, which is connected to P1 and P2 (Figure 1). In particular, at low concentration strontium occupies preferentially M(4) and M(3) sites, whereas at higher concentration it also substitutes calcium in M(1) and M(2), but not in M(5).

The comparison among the ^31^P MAS NMR spectrum of β-TCP and those of the ZnTCP and SrTCP samples confirms XRD results.

In particular, ZnTCP spectra (Figure 8) show a low-frequency shift (about 1 ppm) and a drastic narrowing (FWHM from ~530 to ~195 Hz) of the P2 signal at 4.9 ppm. The P3 signal, at 0.3 ppm, though being less affected, moves toward higher frequencies to 0.6 ppm.

In the case of the Sr substitution (Figure 9), a more complex peak evolution is observed: for low Sr contents (up to 20%) the spectrum gets simpler and is characterized by four relatively broad peaks centered at 5.2 (P2), 1.9 (P2), 0.7 (P1 and P3) and −0.8 (P1 and P3) ppm. At higher Sr contents, the spectrum changes significantly with a severe broadening in agreement with the XRD analysis (see above) which suggests the Sr substitution on all calcium sites but M(5).

These data concerning zinc and strontium substituted TCP, in the whole are in good agreement with those reported by Grigg et al. (2014) for β-TCP doped with aluminum, gallium and sodium, which showed that that ions bigger than calcium (Na) preferentially enter site (4), and those with shorter radius (Al, Ga) substitute calcium at site (5) [42].

### 3.3. ATR-FTIR Spectroscopy

**Figure 10:**
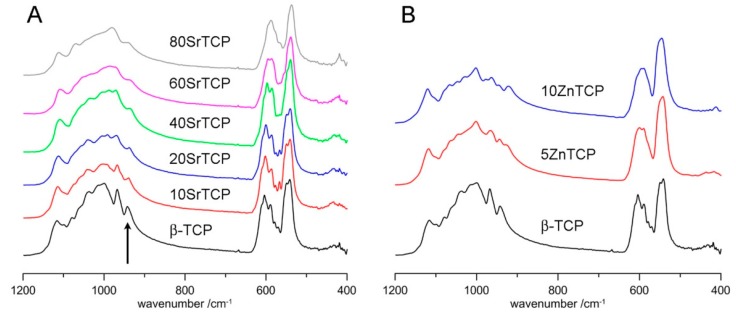
ATR-FTIR spectra of β-TCP at different Sr (**A**) and Zn (**B**) content. The arrow indicates the ν1 band at 943 cm^−1^ in the spectrum of β-TCP.

The infrared absorption spectrum of β-TCP shows a number of bands due to the vibration of the phosphate groups. Infrared and Raman studies identified the bands due to the symmetric stretching ν1 (940–980 cm^−1^), the triple-degenerate asymmetric stretching ν3 (1000–1100 cm^−1^), and the double and triple-degenerate bending ν2 (400–500 cm^−1^) and ν4 (550–600 cm^−1^) [43,44,45]. The infrared absorption spectrum of pure β-TCP is compared with those of samples at increasing Sr content in Figure 10A.

The comparison shows that Sr substitution provokes an increasing degeneracy and a general shift of the bands towards lower wavenumbers. This is particularly evident for the ν1 band which shifts from 943 cm^−1^ in the spectrum of β-TCP to 936 cm^−1^ in the sample at the highest Sr content (Table S1). In the Raman spectrum of β-TCP this band was ascribed to P(1), the band at about 968 cm^−1^ was assigned to P(3), whereas the intermediate band, not appreciable in the ATR-FTIR spectrum, was assigned to P(2) [46,47]. P(1) and P(2) are close to Ca(4); in particular P(1) and P(2) tetrahedra share a common face and an edge, respectively, with Ca(4) site (Figure 1), whereas P(3) is not in the close vicinity of Ca(4). It follows that the shift of the 943 cm^−1^ band at lower wavenumbers on increasing Sr substitution is consistent with its preferential occupancy of Ca(4) site.

Incorporation of zinc into β-TCP structure induces a significantly increased degeneracy of the infrared absorption bands (Figure 10B), in agreement with previously reported data [9,26], which suggests an increasing structural disorder.

## 4. Conclusions

Β-TCP samples were prepared at increasing contents of zinc or strontium. XRD data show that Zn-TCP can be prepared as a single phase up to a zinc content of about 10 at%, whereas a much wider range of substitution, up to about 80 at%, has been obtained for strontium. The results of Rietveld refinements indicate that Zn occupies preferentially the octahedral Ca(5) site, in agreement with the results reported by Kannan et al. (2009) [26] and by Kawabata et al. (2011) [27], whereas it does not show any preference for the Ca(4) site as previously suggested [29]. Zinc substitution provokes a reduction of the cell parameters, a shift of the solid-state ^31^P NMR resonances of the two phosphate close to Ca(5), namely P(2) and P(3), and a general disorder of β-TCP structure as shown by the broadening of the ATR-FTIR bands. In contrast, the relatively small Ca(5) site is not appreciated by strontium, which does not occupy it even at the highest concentrations: after filling the Ca(4) site, strontium occupies Ca(1), Ca(2) and Ca(3), but not Ca(5). The clear preference of strontium for the Ca(4) site, not only at a small content as previously reported [31,32,39] but also at a relatively high content, is further confirmed by the significant shift of the infrared symmetric stretching band at 943 cm^−1^ due to P(1), that is the phosphate more involved into Ca(4) coordination. The preference is also maintained for a strontium content of about 80 at%, even if the high amount of strontium provokes a slight modification the β-TCP structure into the more symmetric β’-TCP. These results provide further, more detailed, information about the influence played by ionic substitution on β-TCP structure, which is of particular interest in the case of strontium and zinc in view of their important biological roles.

## Figures and Tables

**Table 1:** Lattice parameters of β-TCP in samples synthesized at different strontium or zinc content.

	β-TCP	10SrTCP	20SrTCP	40SrTCP	60SrTCP	80SrTCP ^1^	5ZnTCP	10ZnTCP
*a* (Å)	10.4387 (1)	10.4578 (2)	10.4897 (2)	10.5773 (2)	10.6525 (4)	10.6996 (2)	10.3820 (3)	10.3364 (2)
*c* (Å)	37.3979 (6)	37.421 (1)	37.599 (1)	38.189(1)	38.708 (2)	39.226 (1)	37.247 (1)	37.1262 (9)
V (Å^3^)	3529	3543	3583	3700	3804	3889	3477	3435

^1^ Parameters based on β’-TCP setting are: *a* = 10.6996(2) Å, *c* = 19.613(1) Å, V = 1945 Å^3^.

**Table 2:** Refined structural parameters for SrTCP samples.

	M ^1^	10SrTCP		20SrTCP		40SrTCP		60SrTCP		80SrTCP			80SrTCP ^5^	
OF Sr *atoms/cell*														
M(1)	18	0.01	*0*	0.10	*1.8*	0.34	*6.1*	0.62	*11.2*	0.73	*13.1*	M(1)	0.85	*30.6*
M(2)	18	0.05	*0.9*	0.09	*1.6*	0.33	*6.0*	0.68	*12.2*	0.83	*15.0*	M(31)	0.23	*8.28*
M(3)	18	0.17	*3.06*	0.40	*7.2*	0.68	*12.2*	0.67	*12.1*	0.80	*14.4*	M(32)	0.23	*8.28*
M(4) ^2^	6	0.32	*1.92*	0.40	*2.4*	0.41	*2.4*	0.41	*2.4*	0.43	*2.6*	M(4)	0.22	*2.64*
M(5)	6	0	*0*	0	*0*	0.01	*0*	0.01	*0*	0.01	*0*	M(5)	0	*0*
Sr at./cell ^3^		*5.88*		*13.0*		*26.7*		*37.9*		*45.1*			49.8	
Sr at% ^4^		9.4		20.6		42.2		60.0		71.4			79.0	
Rwp (%)		6.3		6.6		12.7		13.8		17.9			9.2	

^1^ multiplicity of crystal site; ^2^ the overall content of site 4 is 0.43; ^3^ total metal atoms inside unit cell are 63.2 [15]; ^4^ from refinement; ^5^ data based on β’-TCP setting [32]. Since β’-TCP cell is half of β-TCP cell, the data of atom/cell have been doubled in order to allow direct comparison with other samples. In β’-TCP M(1) and M(31) + M(32) are equivalent in β-TCP to M(1) + M(2) and M(3) respectively.

**Table 3:** Refined structural parameters for ZnTCP samples.

	M ^1^	5ZnTCP		10ZnTCP	
		OF Sr *atoms/cell*			
M(1)	18	0.05	*0.9*	0.03	*0.5*
M(2)	18	0.07	*1.3*	0.07	*1.2*
M(3)	18	0.07	*1.3*	0.08	*1.4*
M(4) ^2^	6	0.05	*0.0*	0.03	*0.0*
M(5)	6	0.15	*0.9*	0.97	*5.8*
Zn at/cell ^3^	-	*4.4*		*8.9*	
Zn at% ^4^	-	7.0		14.1	
Rwp (%)	-	7.4		8.9	

^1^ multiplicity of crystal site; ^2^ the overall content of site 4 is 0.43; ^3^ total metal atoms inside unit cell are 63.2 [15]; ^4^ from refinement.

## Supplementary Materials

The following are available online at https://www.mdpi.com/2079-4983/10/2/20/s1, Figure S1: XRD patterns of β-TCP-C, β-TCP and calculated pattern; Figure S2: observed and calculated XRD patterns of SrTCP samples; Figure S3: observed and calculated XRD patterns of 80SrTCP, refinement performed using β’-TCP setting; Figure S4: comparison between refinements carried out in β’-TCP and β-TCP settings for sample 60SrTCP; Figure S5: observed and calculated XRD patterns of ZnTCP samples; Table S1: ATR-FTIR bands wavenumbers.

## References

[1] Elliott J.C. (1994). Structure and Chemistry of the Apatites and Other Calcium Orthophosphates.

[2] Bigi A., Cojazzi G., Panzavolta S., Ripamonti A., Roveri N., Romanello M., Noris Suarez K., Moro L. (1997). Chemical and structural characterization of the mineral phase from cortical and trabecular bone. J. Inorg. Biochem..

[3] Dorozhkin S.V. (2016). Multiphasic calcium orthophosphate (CaPO_4_) bioceramics and their biomedical applications. Ceram. Int..

[4] Boanini E., Gazzano M., Bigi A. (2010). Ionic substitutions in calcium phosphates synthesized at low temperature. Acta Biomater..

[5] Shepherd J.H., Shepherd D.V., Best S.M. (2012). Substituted hydroxyapatites for bone repair. J. Mater. Sci. Mater. Med..

[6] Bigi A., Boanini E., Gazzano M., Aparicio C., Ginebra M.P. (2015). Ion substitution in biological and synthetic apatites. Biomineralization and Biomaterials, Fundamentals and Applications.

[7] Šupová M. (2015). Substituted hydroxyapatites for biomedical applications: A review. Ceram. Int..

[8] Bigi A., Boanini E. (2017). Functionalized biomimetic calcium phosphates for bone tissue repair. J. Appl. Biomater. Funct. Mater..

[9] Bigi A., Foresti E., Gandolfi M., Gazzano M., Roveri N. (1997). Isomorphous substitutions in β-tricalcium phosphate: The different effects of zinc and strontium. J. Inorg. Biochem..

[10] Mayer I., Cuisinier F.J.G., Gdalya S., Popov I. (2008). TEM study of the morphology of Mn^2+^-doped calcium hydroxyapatite and β-tricalcium phosphate. J. Inorg. Biochem..

[11] Kannan S., Lemos I.A.F., Rocha J.H.G., Ferreira J.M.F. (2005). Synthesis and characterization of magnesium substituted biphasic mixtures of controlled hydroxyapatite/β-tricalcium phosphate ratios. J. Solid State Chem..

[12] Li X., Ito A., Sogo Y., Wang X., LeGeros R.Z. (2009). Solubility of Mg-containing β-tricalcium phosphate at 25°C. Acta Biomater..

[13] Frasnelli M., Sglavo V.M. (2016). Effect of Mg^2+^ doping on beta–alpha phase transition in tricalcium phosphate (TCP) bioceramics. Acta Biomater..

[14] Zhang M., Wu C., Li H., Yuen J., Chang J., Xiao Y. (2012). Preparation, characterization and in vitro angiogenic capacity of cobalt substituted β-tricalcium phosphate ceramics. J. Mater. Chem..

[15] Yashima M., Sakai A., Kamiyama T., Hoshikawa A. (2003). Crystal structure analysis of β-tricalcium phosphate Ca_3_(PO_4_)_2_ by neutron powder diffraction. J. Solid State Chem..

[16] Bonnelye E., Chabadel A., Saltel F., Jurdic P. (2008). Dual effect of strontium ranelate: Stimulation of osteoblast differentiation and inhibition of osteoclast formation and resorption in vitro. Bone.

[17] Salamanna F., Giavaresi G., Parrilli A., Torricelli P., Boanini E., Bigi A., Fini M. (2019). Antiresorptive properties of strontium substituted and alendronate functionalized hydroxyapatite nanocrystals in an ovariectomized rat spinal arthrodesis model. Mater. Sci. Eng. C-Mater. Biol. Appl..

[18] Shepherd D.V., Kauppinen K., Brooks R.A., Best S.M. (2014). An in vitro study into the effect of zinc substituted hydroxyapatite on osteoclast number and activity. J. Biomed. Mater. Res. Part A.

[19] Sutha S., Karunakaran G., Rajendran V. (2013). Enhancement of antimicrobial and long-term biostability of the zinc-incorporated hydroxyapatite coated 316 L stainless steel implant for biomedical application. Ceram. Int..

[20] Yamada Y., Ito A., Kojima H., Sakane M., Miyakawa S., Uemura T., LeGeros R.Z. (2008). Inhibitory effect of Zn^2+^ in zinc-containing β-tricalcium phosphate on resorbing activity of mature osteoclasts. J. Biomed. Mater. Res. Part A.

[21] Roy M., Bose S. (2012). Osteoclastogenesis and osteoclastic resorption of tricalcium phosphate: Effect of strontium and magnesium doping. J. Biomed. Mater. Res. Part A.

[22] Roy M., Fielding G.A., Bandyopadhyay A., Bose S. (2013). Effects of zinc and strontium substitution in tricalcium phosphate on osteoclast differentiation and resorption. Biomater. Sci..

[23] Chou J., Hao J., Hatoyama H., Ben-Nissan B., Milthorpe B., Otsuka M. (2013). The therapeutic effect on bone mineral formation from biomimetic zinc containing tricalcium phosphate (ZnTCP) in zinc-deficient osteoporotic mice. PLoS ONE.

[24] Boanini E., Torricelli P., Sima F., Axente E., Fini M., Mihailescu I.N., Bigi A. (2018). Gradient coatings of strontium hydroxyapatite/zinc β-tricalcium phosphate as a tool to modulate osteoblast/osteoclast response. J. Inorg. Biochem..

[25] Salamanna F., Giavaresi G., Contartese D., Bigi A., Boanini E., Parrilli A., Lolli R., Gasbarrini A., Barbanti Brodano G., Fini M. (2019). Effect of strontium substituted β-TCP associated to mesenchymal stem cells from bone marrow and adipose tissue on spinal fusion in healthy and ovariectomized rat. J. Cell. Physiol..

[26] Kannan S., Goetz-Neunhoeffer F., Neubauer J., Ferreira J.M.F. (2009). Synthesis and structure refinement of zinc-doped β-tricalcium phosphate powders. J. Am. Ceram. Soc..

[27] Kawabata K., Yamamoto T., Kitada A. (2011). Substitution mechanism of Zn ions in β-tricalcium phosphate. Phys. B.

[28] Matsunaga K., Kubota T., Toyoura K., Nakamura A. (2015). First-principles calculations of divalent substitution of Ca(2+) in tricalcium phosphates. Acta Biomater..

[29] Gomes S., Nedelec J.M., Jallot E., Sheptyakov D., Renaudin G. (2011). Unexpected mechanism of Zn^2+^ insertion in calcium phosphate bioceramics. Chem. Mater..

[30] Kannan S., Pina S., Ferreira J.M.F. (2006). Formation of strontium-stabilized β-tricalcium phosphate from calcium-deficient apatite. J. Am. Ceram. Soc..

[31] Nandha Kumar P., Boovarasan M., Singh R.K., Kannan S. (2013). Synthesis, structural analysis and fabrication of coatings of the Cu_2+_ and Sr_2+_ co-substitutions in β-Ca_3_(PO_4_)_2_. RSC Adv..

[32] Belik A.A., Izumi F., Stefanovich S.Y., Malakho A.P., Lazoryak B.I., Leonidov I.A., Leonidova O.N., Davydov S.A. (2002). Polar and centrosymmetric phases in solid solutions Ca_3-x_Sr_x_(PO_4_)_2_ (0 ≤ x ≤ 16/7). Chem. Mater..

[33] Lutterotti L. (2010). Total pattern fitting for the combined size-strain-stress-texture determination in thin film diffraction. Nucl. Instrum. Meth. B.

[34] Mellier C., Fayon F., Schnitzler V., Deniard P., Allix M., Quillard S., Massiot D., Bouler J.M., Bujoli B., Janvier P. (2011). Characterization and properties of novel gallium-doped calcium phosphate ceramics. Inorg. Chem..

[35] Segall M.D., Lindan P.J.D., Probert M.J., Pickard C.J., Hasnip P.J., Clark S.J., Payne M.C. (2002). First-principles simulation: Ideas, illustrations and the CASTEP code. J. Phys.-Condensed Matter.

[36] Clark S.J., Segall M.D., Pickard C.J., Hasnip P.J., Probert M.J., Refson K., Payne M.C. (2005). First principles methods using CASTEP. Z. Kristallogr..

[37] Young R.A. (1993). The Rietveld Method.

[38] Nord A.G. (1983). Incorporation of divalent metals in whitlockite-related β-Ca_3_(PO_4_)_2_. Neues Jahrbuch fuer Mineralogie Monatshefte.

[39] Kannan S., Goetz-Neunhoeffer F., Neubauer J., Pina S., Torres P.M.C., Ferreira J.M.F. (2010). Synthesis and structural characterization of strontium and magnesium co-substituted β-tricalcium phosphate. Acta Biomater..

[40] Bigi A., Falini G., Foresti E., Ripamonti A., Gazzano M., Roveri N. (1996). Rietveld structure refinement of synthetic magnesium substituted β-tricalcium phosphate. Z. Kristallogr..

[41] Obadia L., Deniard P., Alonso B., Rouillon T., Jobic S., Guicheux J., Julien M., Massiot D., Bujoli B., Bouler M. (2006). Characterization and properties of novel gallium-doped calcium phosphate ceramics. Chem. Mater..

[42] Grigg A.T., Mee M., Mallinson P.M., Fong S.K., Gan Z., Dupree R., Holland D. (2014). Cation substitution in β-tricalcium phosphate investigated using multi-nuclear, solid-state NMR. J. Solid. State Chem..

[43] Jillavenkatesa A., Condrate R.A. (1998). The infrared and Raman spectra of β- and α-tricalcium phosphate (Ca_3_(PO_4_)_2_). Spectrosc. Lett..

[44] De Aza P.N., Santos C., Pazo A., De Aza S., Cusco R., Artus L. (1997). Vibrational properties of calcium phosphate compounds. 1. Raman spectrum of β-tricalcium phosphate. Chem. Mater..

[45] De Aza P.N., Guitian F., Santos C., De Aza S., Cusco R., Artus L. (1997). Vibrational properties of calcium phosphate compounds. 2. Comparison between hydroxyapatite and β-tricalcium phosphate. Chem. Mater..

[46] Jakeman R.J.B., Cheetham A.K., Clayden N.J., Dobson C.M. (1989). A magic angle spinning NMR study of the phase diagram Ca_3-x_Zn_x_(PO_4_)_2_. J. Solid State Chem..

[47] Quillard S., Paris M., Deniard P., Gildenhaar R., Berger G., Obadia L., Bouler J.M. (2011). Structural and spectroscopic characterization of a series of potassium and/or sodium-substituted β-tricalcium phosphate. Acta Biomater..

