# Supplementary material for: Strontium and Zinc Substitution in β-Tricalcium Phosphate: An X-ray Diffraction, Solid State NMR and ATR-FTIR Study
Source: J Funct Biomater. 2019 May 5;10(2):20. doi: 10.3390/jfb10020020 (PMC6616520; doi:10.3390/jfb10020020)
Supplement: Supplementary file 1 [file jfb-10-00020-s001.pdf]

Supplementary Information

Strontium and zinc substitution in β-tricalcium phosphate: an X-ray diffraction, solid state NMR and ATR-FTIR study

Elisa Boanini, Massimo Gazzano, Carlo Nervi, Michele R. Chierotti, Katia Rubini, Roberto Gobetto and Adriana Bigi

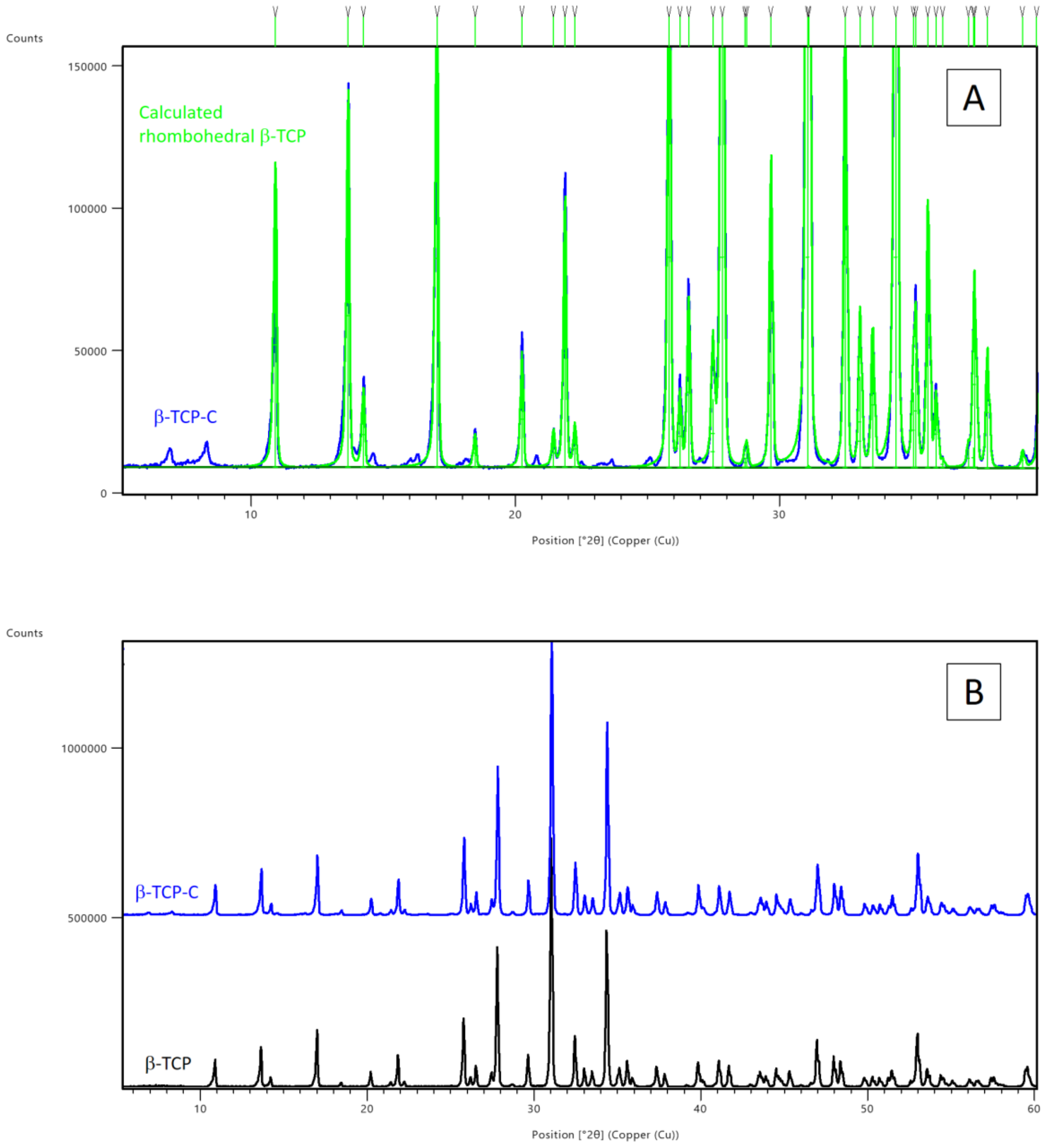

Figure S1: (A) comparison between XRD pattern of β-TCP-C (blue) and the calculated pattern for rhombohedral β-TCP (green); (B) comparison of β-TCP-C and β-TCP XRD scans.

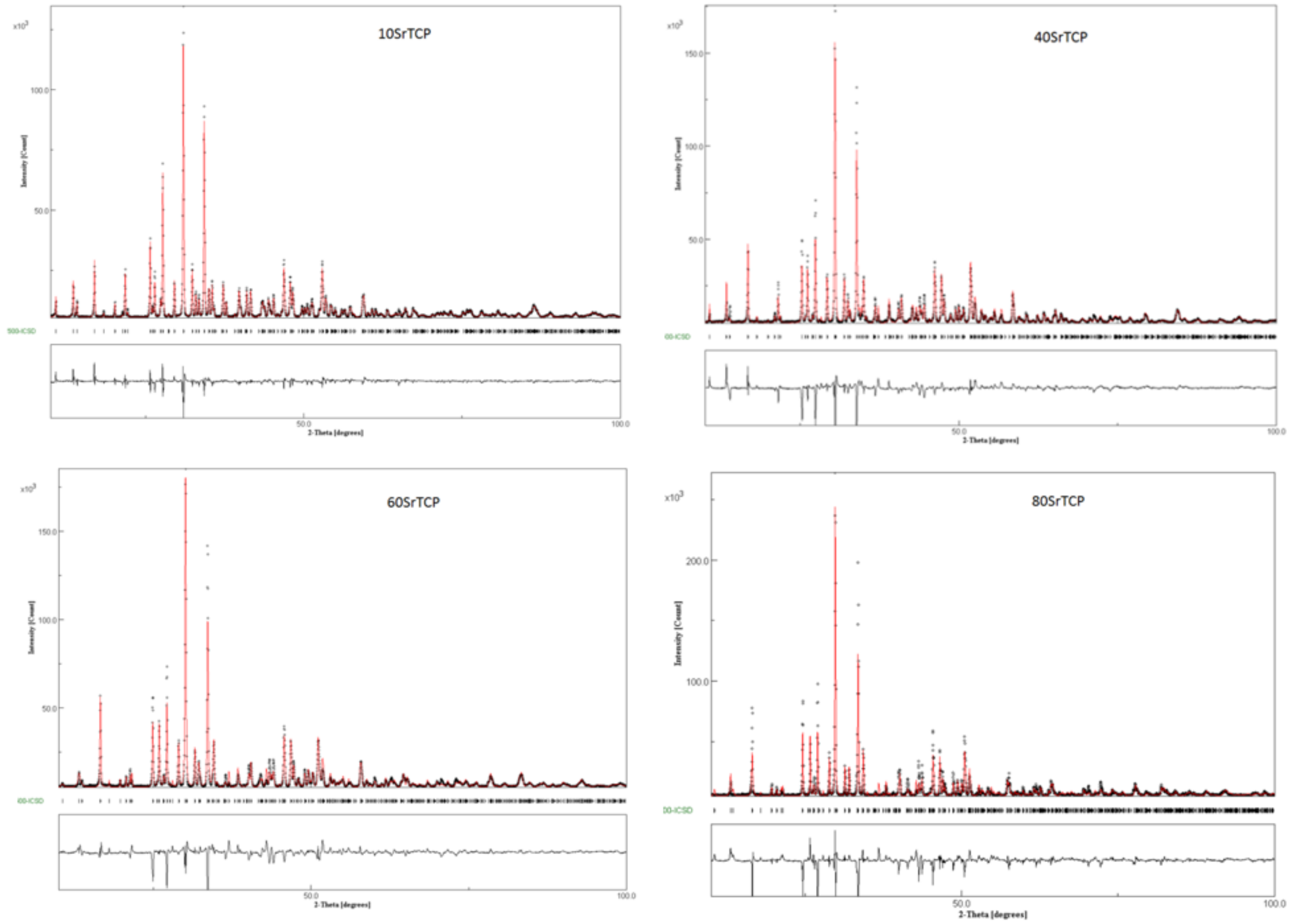

Figure S2: comparison of the observed (dots) and calculated (red) XRD patterns of 10SrTCP, 40SrTCP, 60SrTCP, 80SrTCP. Refinements carried out on the β-TCP setting. At the bottom reflection markers and curve difference.

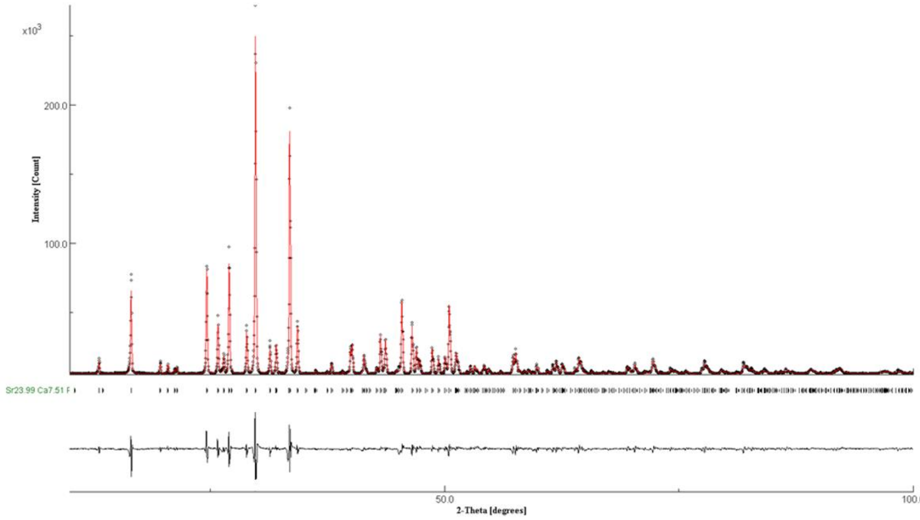

Figure S3: comparison of the observed (dots) and calculated (red) XRD patterns of 80SrTCP. Refinement carried out on the β'-TCP setting. At the bottom reflection markers and difference curve.

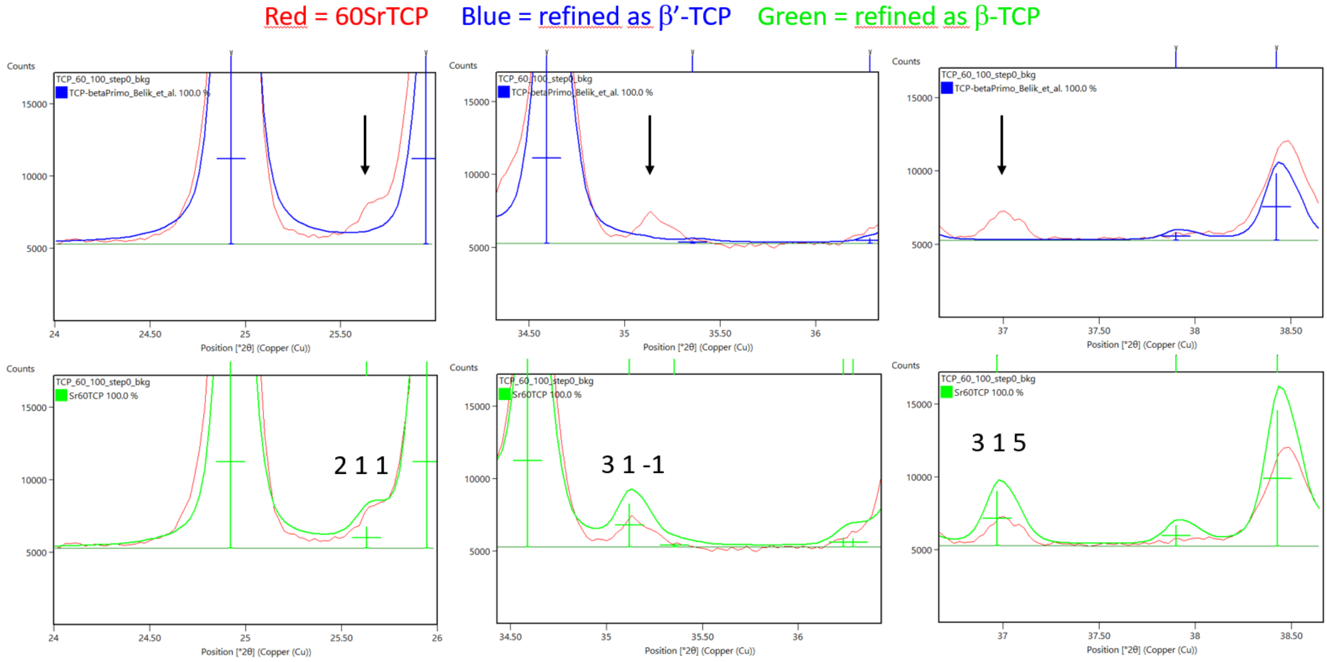

Figure S4: comparison between refinements carried out in β′-TCP (top) and β-TCP (bottom) settings for sample 60SrTCP. Arrows show reflections not matched in the β′-TCP structure (top); *h k l* indexes assigned in β-TCP structure are indicated (bottom).

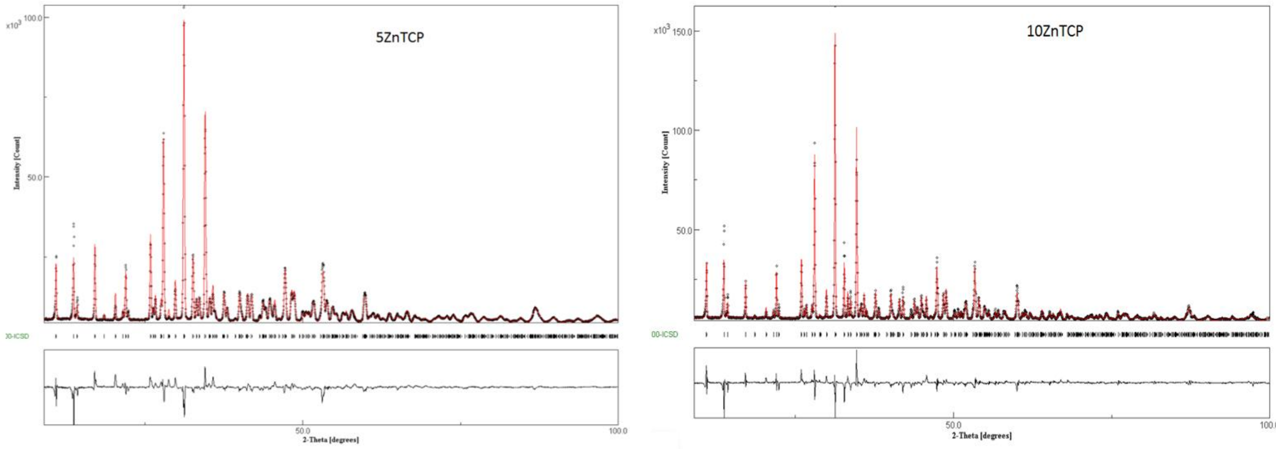

Figure S5: comparison of the observed (dots) and calculated (red) XRD patterns of 5ZnTCP and 10ZnTCP. At the bottom reflection markers and curve difference.

Table S1: ATR-FTIR bands wavenumbers (cm^{-1}).

	β–TCP	10SrTCP	20SrTCP	40SrTCP	60SrTCP	80SrTCP
ν3	1116	1114	1112	1109	1108	1112
	1078	1075			1053	1070
	1037	1041	1039	1036	1033	
	1000	1002	1005	1006	984	980
ν1	968	967	970	971		
	943	940	939	937	936	936
ν4	604	602	600	597	595	
	589	587	586	586	586	588
	579	577	575			
	570	567	566			567
	550	550	550			
	541	541	541	540	539	537
